# Characterization of colistin tissue pharmacokinetics by microdialysis

**DOI:** 10.1186/2050-6511-13-S1-A74

**Published:** 2012-09-17

**Authors:** Peter Matzneller, William Couet, Patrice Gobin, Markus Müller, Markus Zeitlinger

**Affiliations:** 1 Department of Clinical Pharmacology, Medical University of Vienna, 1090 Vienna, Austria; 2 Inserm ERI-23, Faculty of Medicine, University of Poitiers, 86022 Poitiers Cedex, France

## Background

Colistin is an important antimicrobial treatment option against multidrug-resistant Gram-negative bacteria. However, colistin is a large and chemically complex molecule and information on its ability to penetrate into tissues remains sparse. Thus, the present work investigated the ability of microdialysis (µD) to assess pharmacokinetics (PK) of colistin in the interstitium of soft tissues, i.e. at a potential site of infection.

## Methods

*In vitro:* Colistin recovery for linear CMA 66 µD probes with a molecular weight cut-off of 100 kDa was assessed through forward and reverse µD for different colistin concentrations. *In vivo:* Three male healthy volunteers received a single intravenous dose of 2.5 million international units of the inactive prodrug colistin methanesulfonate. Colistin concentrations in plasma and in µD samples obtained from two probes inserted into subcutaneous adipose tissue of the thigh were determined. Retrodialysis was used for probe calibration. In both settings, µD was performed with and without addition of albumin to perfusion solutions and colistin was quantified by liquid chromatography/tandem mass spectrometry (LC-MS/MS).

## Results

*In vitro*, colistin recovery was constant over time and showed mean recovery values of 52 ± 3 and 71 ± 8% for forward and reverse µD, respectively. *In vivo*, recovery of colistin was 43 ± 15%. In both settings, colistin recovery was not improved by addition of albumin to µD perfusion solutions. Due to small volumes, reliable quantification of colistin was not possible in some µD samples, yet maximum concentrations in adipose tissue were relatively high (0.76 ± 0.21 µg/mL) compared with those in plasma (1.2 ± 0.43 µg/mL) attesting for extra-vascular distribution.

## Conclusions

The present data demonstrate the feasibility of µD for evaluation of colistin tissue pharmacokinetics and show opportunities for optimization of experimental setting.

